# Multianalytical Method Validation for Qualitative and Quantitative Analysis of Solvents of Abuse in Oral Fluid by HS-GC/MS

**DOI:** 10.1155/2016/1029286

**Published:** 2016-05-04

**Authors:** Bruna Claudia Coppe, Bruna Tassi Borille, Taís Regina Fiorentin, Ana Laura Bemvenuti Jacques, Ana Claudia Fagundes, Stela Maris de Jezus Castro, Lysa Silveira Remy, Flavio Pechansky, Renata Pereira Limberger

**Affiliations:** ^1^Postgraduate Program in Pharmaceutical Sciences, School of Pharmacy, Federal University of Rio Grande do Sul, Avenida Ipiranga 2752, Santana, 90610-000 Porto Alegre, RS, Brazil; ^2^Department of Statistics, Federal University of Rio Grande do Sul, Avenida Bento Gonçalves 9500, 91509-200 Porto Alegre, RS, Brazil; ^3^Center for Drug and Alcohol Research, Federal University of Rio Grande do Sul, Rua Ramiro Barcelos 2350, Santana, 90035-903 Porto Alegre, RS, Brazil

## Abstract

The use of oral fluid as a biological matrix to monitor the use of drugs of abuse is a global trend because it presents several advantages and good correlation to the blood level. Thus, the present work aimed to develop and validate an analytical method for quantification and detection of solvents used as inhalants of abuse in oral fluid (OF), using Quantisal*™* as collector device by headspace and gas chromatography coupled with a mass detector (HS-GC/MS). Chromatographic separation was performed with a ZB-BAC1 column and the total time of analysis was 11.8 min. The method showed good linearity (correlation coefficient higher than 0.99 for all solvents). The limits of detection ranged from 0.05 to 5 mg/L, while the lower limits of quantification ranged from 2.5 to 12.5 mg/L. Accuracy, precision, matrix effect, and residual effect presented satisfactory results, meeting the criteria accepted for the validation of bioanalytical methods. The method showed good selectivity considering that, for solvents coeluting at the same retention time, resolution was performed by the mass detector. The method developed proved to be adequate when applied in OF samples from users of drugs and may be used to monitor the abuse of inhalants in routine forensic analyses.

## 1. Introduction

The abuse of inhalants, also known as abuse of volatile substances or abuse of solvents [1, 2], is a global issue with major consequences for users, their families, and society. It is defined as the intentional inhalation of volatile substances, aiming to reach an altered mental state [1–4]. This problem has been neglected in researches on drug abuse and in national and international drug policies [4].

Several commercial products may be used as inhalants, such as glues, paints, varnishes, removers, sprays, nail polishers, and fuels [4, 5]. These products may be classified according to their chemical structure, commercial use, or pharmacological profile [5]. Products containing inhalants are usually a mix of two or more solvents. Toluene is present in many products for household and industrial use and is most often used as a solvent inhalant abuse worldwide [5–7]. In Brazil, the main products used with the presence of solvents are* lança-perfume* (chloroethane) and* cheirinho-da-loló*—a homemade version of* lança-perfume* containing a mix of diethyl ether, ethanol, and chloroform [6, 7]. Recently, another formula based on Freon*™* gases (gas based on halogenated substances) has been intentionally used mainly at electronic music parties, and it is called “success” and “the drug of success.” Freon is a refrigerant gas based on halogenated substances used in home appliances such as freezers and air conditioners [8, 9], and it has been applied as raw material in the fabrication of inhalants of abuse. Although data on inhalants are abundant in literature, there is still little information on the abuse of Freon [9].

The abuse of these substances is a disseminated problem among children, adolescents living in the streets [10, 11], and rave partygoers because of its psychoactive effects [12]. Inhalants are featured as the fourth drug of abuse most used in Brazil, after alcohol, marijuana, and tobacco [10–12]. As for the United States, studies show that approximately 100 youngsters die per year due to cardiac arrest associated with the use of inhalants [10–12].

Inhalants may be administered by several methods [2, 4]. Glues are usually placed in either plastic or paper bags so their vapors are inhaled through the mouth and nose (bagging); pieces of fabric may be soaked with solvents and inhaled (huffing); they may also be inhaled directly from their recipients (sniffing) or by direct spraying in the mouth or nose (dusting) [2, 4, 13, 14]. The higher inhalant concentration may be achieved through bagging and huffing; therefore, these methods of abuse are mostly preferred [2, 15].

The use of inhalants allows high concentrations to quickly reach lungs and brain [16]. The toxic effects resulting from inhalation are similar to the ones caused by ethanol; first, the users feel euphoric and uninhibited followed by lethargy, slurred speech, and other depressant effects of the central nervous system (CNS) [16, 17]. High doses may cause convulsions, asphyxia, cardiac dysfunction, cessation of breathing, coma, and death [16–18].

Considering the strong consumption of inhalant solvents, it is important to define a laboratory parameter to assess the acute exposure to these solvents. Therefore, gas chromatography coupled with mass detector (GC/MS) may be considered the method of choice to identify and separate solvents of abuse, mostly when associated with the headspace (HS) sampling technique. Although GC/MS is considered the “gold standard” technique for unequivocal confirmation of results and is present in forensic laboratories, most studies found in literature use gas chromatography coupled with flame ionization detector (GC/FID) [18–22].

Thus, the present work aims to develop and validate an analytical method by HS-GC/MS for simultaneous detection and quantification of solvents in oral fluid (OF). The OF biological matrix was chosen because it presents as an advantage the possibility of a quick, noninvasive collection, under direct supervision [23]. Although blood is the most used matrix to assess the exposure to volatile compounds, the suggestion of using OF as biological matrix to monitor the use of drugs of abuse is a global trend because it presents good correlation to blood level [23, 24], and it may be collected through commercial devices [24]. Quantisal was the device chosen to collect OF samples, for its best collection volume indicator (1 mL) and because it has already been validated by our research group [24, 25]. After the method was validated, it was applied in OF samples from 22 volunteers, men and women, who were users of multiple drugs, and participants of the Assistance Program for Users of “Club Drugs,” in partnership with the Psychiatric Service of the Clinics Hospital of Porto Alegre and the Center for Drugs and Alcohol Research (CPAD) of the Federal University of Rio Grande do Sul (UFRGS), Brazil.

## 2. Materials and Methods

### 2.1. Reagents and Materials

Ethanol, diethyl ether, dichloromethane, chloroform, ethyl acetate, n-butanol, n-propanol, toluene, xylene, and isopentanol (internal standard, IS) were obtained from Tedia Company (Fairfield, OH, USA). An analytical standard Freon Mix containing a mix of dichlorodifluoromethane (Freon 12), 1,2-dichloro-1,1,2,2-tetrafluoroethane (Freon 114), dichlorofluoromethane (Freon 21), chlorodifluoromethane (Freon 22), and trichlorofluoromethane (Freon 11) in ethyl acetate solution was obtained from Sigma-Aldrich. Quantisal OF collection devices, filters, and a preservative buffer solution were obtained from Immunalysis Corporation (Pomona, CA, USA). Each device contains a collector that turns blue when about 1 mL of OF is gathered and a plastic tube for transportation with 3 mL of preservative buffer, resulting in a final sample volume of 4 mL. Headspace vials and aluminum screw caps with PTFE-silicone septa were obtained from Agilent Technologies (Agilent J&W Scientific, Folsom, CA, USA).

### 2.2. Biological Samples

OF samples free of volatile solvents from six volunteers were used for method validation. Samples were stored in a freezer until the moment of analyses.

For the application of the method developed, OF samples were collected from participating volunteers of an Assistance Program to Drugs Users. The OF samples from the volunteers were collected at the outpatient Psychiatric Service of the Clinics Hospital of Porto Alegre. Ethical approval was given by the Ethics Committee of the Clinics Hospital of Porto Alegre.

### 2.3. Preparation of Working Solutions and Internal Standard

The working solutions of ethanol, diethyl ether, dichloromethane, chloroform, ethyl acetate, and n-butanol were prepared in methanol with a concentration of 2000 mg/L. Isopentanol (IS) was prepared with a concentration of 2000 mg/L. After preparation, all solutions were stored in a freezer until the moment of use.

### 2.4. Sample Preparation

The substances were grouped according to the working concentration range (Table 1). To build the calibration curve, proper dilutions were performed until reaching seven concentrations, which were added to 1 mL of blank OF. The calibration curve of Group A was built in the range of 50 mg/L to 1000 mg/L and for Group B in the range of 10 mg/L to 120 mg/L. The LLOQ (lower limit of quantification), LQC (low quality control), MQC (medium quality control), HQC (high quality control), and DQC (dilution quality control) were prepared in concentrations of 50, 100, 500, 800, and 500 mg/L, respectively, for Group A, and 10, 20, 60, 100, and 50 mg/L, respectively, for Group B.

Final solutions were diluted with 3 mL of Quantisal preservative so to mimic the process using the Quantisal collection device and then vortexed for 10 seconds. Next, an aliquot of 1 mL was removed and transferred to a 10 mL headspace vial. Ten *μ*L of IS solution was added and the vial was immediately sealed and placed in the vial rack of the autosampler, so to be submitted to chromatographic analysis.

### 2.5. Conditions of HS-GC/MS

Analyses were performed with a GC 5975C chromatograph coupled with a 7890A mass detector (Agilent Technologies, CA, USA) and equipped with HS automatic injector (CTC Analytics Combipal, Basel, Switzerland). The column used for method validation was the ZB-BAC1, Zebron (Phenomenex) (30 m × 0.32 mm × 1.8 *μ*m) provided by Alcrom (São Paulo, Brazil); also aiming to improve specificity and to compare the order of elution of analytes, a Carbowax column (30 m × 0.25 mm × 0.25 *μ*m) was tested. Oven temperature was programed at 30°C (2.5 min) with ramp rate of 5°C/min to 65°C, followed by a ramp rate of 60°C/min to 200°C, which was maintained for 1 min, with a total time of analysis of 11.8 min. The injector was maintained at 200°C with split ratio of 25 : 1. The temperatures of the transfer line (interface), source, and quadrupole were maintained at 220°C, 230°C, and 150°C, respectively. Ultrapure helium was used as carrier gas at a flow rate of 1.4 mL/min. The mass detector system was operated in electron impact ionization at 70 eV and in SIM (Single Ion Monitoring) mode. The ions monitored for ethanol were* m/z* 45, 46, 31, and 29; for diethyl ether* m/z* 74, 59, 45, and 31; for dichloromethane* m/z* 86, 84, 51, and 49; for chloroform* m/z* 119, 121, 83, and 47; for ethyl acetate* m/z* 88, 61, 45, and 43; for butanol* m/z* 74, 56, 41, and 28; for isopentanol (IS)* m/z* 87, 70, 55, and 42. The ions underlined were used for quantification. Solvent response was assessed by the ratio between peak areas of the analyte and the IS.

Headspace was maintained at 85°C with incubation time of 5 min. Experimental conditions of headspace time and temperature were chosen based on previous studies performed by our group [25, 26].

### 2.6. Method Validation

Validation was performed according to recommendations of the USA Food and Drug Administration (FDA) [27] and the Brazilian Health Surveillance Agency (ANVISA) [28]. The parameters assessed were selectivity, matrix effect, residual effect, linearity, precision, accuracy, limit of detection, limit of quantification, and stability [27, 28].

#### 2.6.1. Selectivity

Selectivity was assessed through the analysis of six OF samples from different individuals who were part of the control group and the results were compared to samples of the lower limit of quantification (LLOQ). Complementarily, other solvents added to the sample were analyzed in order to verify their potential interference on the analysis in the retention times of analytes. The solvents tested were n-propanol, isopropanol, toluene, xylene, and Freon Mix.

#### 2.6.2. Residual Effect and Matrix Effect

In the assessment of residual effect, a blank sample was previously analyzed and two blank samples were analyzed after a sample of the upper limit of quantification (ULOQ). Results were compared to the responses obtained in the LLOQ, and results above 20% in retention time (rt) of analytes and 5% in IS were not accepted.

Matrix effect was assessed through the analysis of three high quality controls (HQC) and three low quality controls (LQC) in matrix and in distilled water. Results were assessed through the matrix effect (ME), and coefficient of variation (CV%) among ME above 15% was not allowed:

(1) ME=response  of  analyte  in  OF/response  of  IS  in  OFresponse  of  analyte  in  solution/response  of  IS  in  solution.

#### 2.6.3. Linearity

The linearity of the concentration range proposed was verified through the construction of three calibration curves in three different days including seven concentrations: Group A: 50, 75, 100, 250, 500, 750, and 1000 mg/L and Group B: 10, 20, 40, 60, 80, 100, and 120 mg/L. These data allowed obtaining the equation of the straight line through linear regression. The suitability of the adjusted models was evaluated by residual analysis, and, for cases where there was heterogeneity of variance, which leads to obtain inconsistent estimates for the regression coefficients, standard errors, we opted for the use of robust estimators for the same. Data analysis was performed using statistical software SAS, version 9.4.

#### 2.6.4. Precision and Accuracy

Precision and accuracy were run in three different days through the analysis of five replicates of LLOQ, LQC, MQC, HQC, and DQC. Precision was assessed through CV (%), and accuracy was assessed through the relative standard deviation (RSD). Accuracy and precision allowed values below 15%, except for the LLOQ, which allowed values below or equal to 20%.

#### 2.6.5. Limit of Detection and Limit of Quantification

The LOQ was determined with precision values below 20% and accuracy values within 80–120% through the analysis of five samples in the concentrations of the LLOQ. The LOD was estimated at a signal/noise ration of three.

#### 2.6.6. Stability

Stability was assessed through the analysis of three LQC and three HQC analyzed after 24 hours, 48 hours, and 10 days stored in freezer and after five freezing and thawing cycles (24 h). The stability of IS and analytes in solution was analyzed. For the study of stability, calibration curves prepared in the day of analysis were used and the results were assessed through the standard deviation of the mean concentrations obtained in relation to the nominal value, not allowing values above 15%.

## 3. Results and Discussion

### 3.1. Method Validation

**Figure 1:**
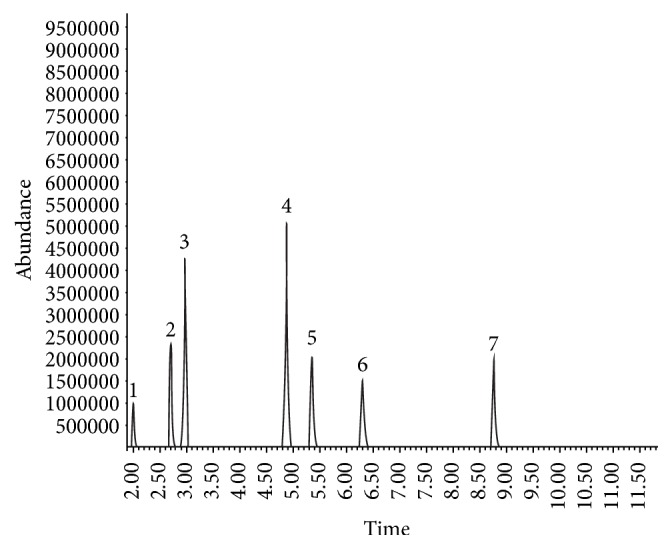
Chromatographic analysis of quantified solvents. (1) Ethanol, (2) diethyl ether, (3) dichloromethane, (4) chloroform, (5) ethyl acetate, (6) n-butanol, and (7) isopentanol/IS.

**Figure 2:**
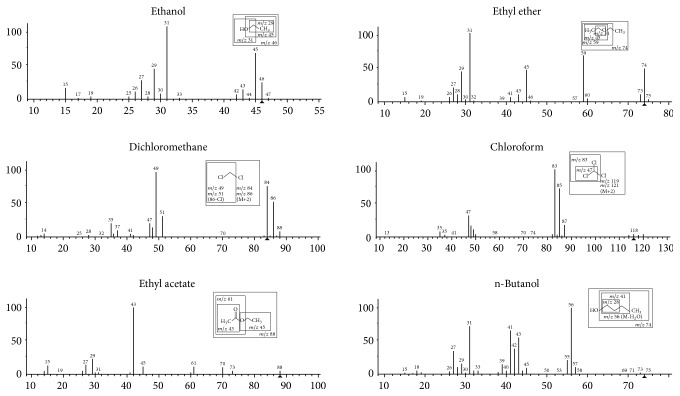
Mass spectra ethanol, diethyl ether, dichloromethane, chloroform, ethyl acetate, and n-butanol with their proposed fragmentation.

The HS-GC/MS method was developed and validated for quantitative analysis of six solvents, which presented proper resolution (Figure 1). Solvents were identified through the retention time and their respective mass spectra in which four ions were monitored for each substance, considering these were the most abundant and representative of each of the molecules (Figure 2).

In order to improve the specificity of the method, all quantified and qualified solvents were injected into a second column (Carbowax), in which polarity was different enough to change retention times and order of elution of the solvents studied; the results are described in Table 2. The use of different columns represents one more analytical parameter that aims to improve the reliability of the result obtained.

#### 3.1.1. Selectivity

**Figure 3:**
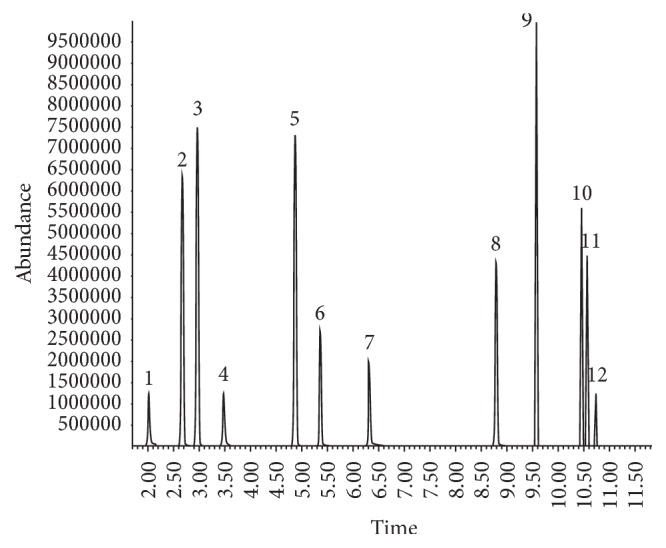
Chromatogram of simultaneous analysis of ten organic solvents for the assessment of selectivity. (1) Ethanol, (2) diethyl ether, (3) dichloromethane, (4) n-propanol, (5) chloroform, (6) ethyl acetate, (7) n-butanol, (8) isopentanol (IS), (9) toluene, (10)* p*-xylene, (11)* m*-xylene, and (12)* o*-xylene.

After analyzing six different OF samples, there were no peaks close to the retention times of analytes. Moreover, selectivity was assessed by analyzing other volatile substances that may be present in products used as inhalants of abuse or used in their adulteration, such as paints, resins, and fuels. Additions to the sample were n-propanol, toluene, and xylene, which coeluted in different retention times from the quantified analytes and the IS (Figure 3).

**Figure 4:**
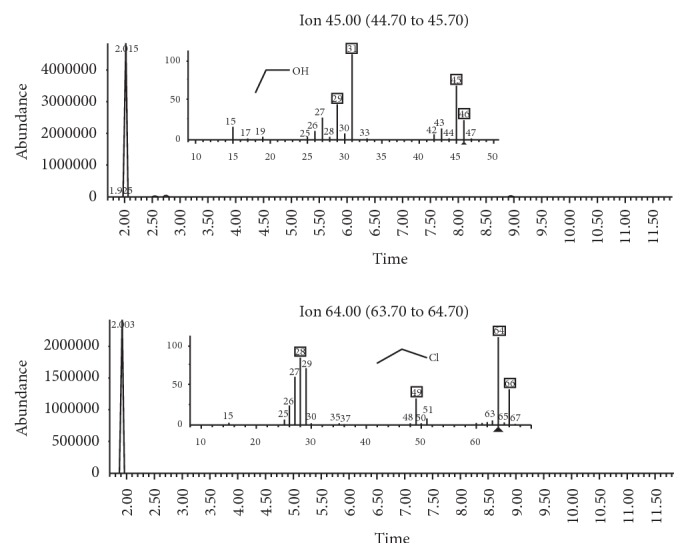
Chromatogram of a simultaneous analysis of ethanol and chloroethane and their mass spectra.

When adding chloroethane, which is the main compound of* lança-perfume*, one of the most popular inhalants of abuse in Brazil [7], it coeluted at the same retention time as ethanol, considering that identification had to be performed through the mass spectrum of these substances, in which spectra were different enough to allow unequivocal identification of each one (Figure 4). Identification and quantification of ethanol and chloroethane by GC/MS excluded the possibility of incorrect results. The analysis by GC/MS allows the identification and quantification of analytes [29], and it is advantageous in comparison to GC/FID, which is mostly studied for the analysis of inhalants. The analysis by GC/FID does not provide structural information of molecules, which prevents confirming the identity of these molecules. The analysis of both solvents becomes imperative, considering that ethanol is not only part of the composition of inhalants of abuse but the main component of alcoholic beverages, providing that the use of inhalants and the consumption of alcoholic beverages may occur simultaneously.

**Figure 5:**
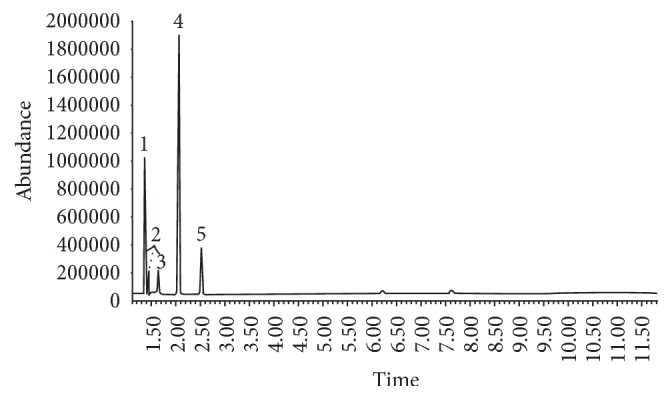
Chromatogram of the analysis of chlorofluorocarbons (Freon Mix). (1) Chlorodifluoromethane, (2) dichlorodifluoromethane, (3) 1,2-dichlorotetrafluoroethane, (4) dichlorofluoromethane, (5) trichlorofluoromethane, and (6) ethyl acetate.

Additionally, a Freon Mix standard was injected, containing a mix of dichlorodifluoromethane (Freon 12), 1,2-dichloro-1,1,2,2-tetrafluoroethane (Freon 114), dichlorofluoromethane (Freon 21), chlorodifluoromethane (Freon 22), and trichlorofluoromethane (Freon 11) in ethyl acetate solution (Figure 5). It was possible to observe that dichlorofluoromethane (Freon 21) coeluted at the same retention time as ethanol, and identification, in this case, should be done by the analysis of mass spectrum of each substance presenting distinct ions.

#### 3.1.2. Residual Effect and Matrix Effect

The response of interference peaks in retention times of analytes on blank samples injected after the upper limit of quantification (ULOQ) was below 5%. The matrix effect was also within the limits accepted, presenting coefficients of variation below 15%, as recommended by the guidelines [27, 28].

#### 3.1.3. Linearity

In the concentration range studies and for all compounds, the analytical response was linear with coefficients of determination (*R* ^2^) higher than 0.99, showing a proportional increase of the peak area ratio in relation to analyte concentration, and a correct adjustment to the linear model was achieved with high significance of regressions for all solvents analyzed (Table 3). Furthermore, the quadratic component was tested in the model and was not significant for all solvents.

#### 3.1.4. Limit of Detection and Limit of Quantification

The lower limit of quantification of the method proposed was 12.5 mg/L for ethanol and butanol and 2.5 mg/L for diethyl ether, dichloromethane, chloroform, and ethyl acetate. The limit of detection estimated by signal/noise ratio of approximately three times was 5 mg/L for ethanol and butanol, 0.1 mg/L for diethyl ether and ethyl acetate, and 0.05 mg/L for dichloromethane and chloroform. These results show the increase in sensitivity of GC/MS when compared to other methods found in literature using GC/FID [22].

#### 3.1.5. Precision and Accuracy

The tests for precision and accuracy, inter- and intraday, presented coefficients of variation (CV) and relative standard deviation (RSD) below 20% for LLOQ and below 15% for the remaining controls, meeting the criteria for validation of bioanalytical methods applied (Table 4).

#### 3.1.6. Stability

The results obtained in the stability study for the period of 24 h, 48 h, and 10 days in freezer (−1 to −8°C) showed deviation below 15% in relation to the nominal concentration for ethanol and butanol. Also for both substances, deviation was below 15% after performing cycles of freezing and thawing. Ethyl acetate was stable for a period of 24 h in freezer, while diethyl ether, dichloromethane, and chloroform were not stable with range above 15%. Diethyl ether, dichloromethane, chloroform, and ethyl acetate showed deviations above 15% after 48 h and 10 days in freezer and after cycles of freezing and thawing. The stability of analytes and IS in solution for 20 h in freezer was attested. Considering that stability test aims to establish ideal conditions of storage and transportation of samples, and regarding volatile substances, it is recommended that they are frozen immediately after collection and analyzed in a period shorter than 24 h to avoid evaporation of solvents in analysis.

### 3.2. Application of the Method Developed

The method developed was applied in participating volunteers from an Assistance Program to Drugs Users as part of a project developed by the Center for Drug and Alcohol Research of the Federal University of Rio Grande do Sul, Brazil.

The collection of OF was performed through Quantisal collection device from twenty-two volunteers who accepted to participate in the study and signed the consent term. After collection, the samples were immediately frozen until the moment of analysis by HS-GC/MS.

From the twenty-two samples collected, thirteen showed the presence of peaks of ethanol. For the remaining solvents, all samples were negative. The results for ethanol quantification in OF ranged from 50 mg/L to 60 mg/L; such concentrations were very close to the limit of quantification. This can be explained by the fact that volunteers selected are participants of an Assistance Program to Drugs, a different situation from the target public. By ethic reasons, this method could not be applied in specific places where the use of these substances is more common, as electronic music parties. Moreover, the stability of these solvents is shorter than ethanol mainly because of the high level of volatility which contributes to a short period of detection.

## 4. Conclusion

The validated method proved to be fast and sensitive, allowing the quantification of low concentrations of ethanol, diethyl ether, dichloromethane, chloroform, ethyl acetate, and n-butanol as well as the qualitative identification of chloroethane, n-propanol, toluene, xylene, and chlorofluorocarbons. The HS technique associated with GC/MS allows an unequivocal analysis of analytes, showing advantages in multianalytical analyses such as the case of inhalants of abuse. OF proved to be a promising biological matrix in the toxicological analysis of inhalants, and the use of Quantisal buffer was adequate for the analysis. The method developed may be used to monitor the abuse of inhalants in routine forensic and clinical analysis.

## Figures and Tables

**Table 1:** Grouping of substances by working concentration range (mg/L).

Analyte	Working concentration range (mg/L)	Code
Ethanol	50–1000	Group A
n-Butanol	50–1000	

Diethyl ether	10–120	Group B
Dichloromethane	10–120	
Chloroform	10–120	
Ethyl acetate	10–120	

**Table 2:** Retention time (min) of the solvents tested in both columns.

Analyte	Retention time (min)		
	ZB-BAC1 column (Phenomenex)	Analyte	Carbowax column (Agilent)
Ethanol	2.005	Diethyl ether	1.459
Diethyl ether	2.691	Ethyl acetate	2.695
Dichloromethane	2.952	Dichloromethane	3.109
n-Propanol	3.465	Ethanol	3.482
Chloroform	4.864	Chloroform	4.624
Ethyl acetate	5.367	Toluene	5.026
n-Butanol	6.293	n-Propanol	5.469
Isopentanol	8.774	*p*-Xylene	7.008
Toluene	9.587	*m*-Xylene	7.212
*p*-Xylene	10.460	*o*-Xylene	7.398
*m*-Xylene	10.571	n-Butanol	8.092
*o*-Xylene	10.726	Isopentanol	9.707

**Table 3:** Results obtained from linear regression.

Analytes	Equation^a^	*R* ^2^ ^b^	*P* ^c^
Ethanol	*y* = 1.2667*x* + 0.0261	0.998	<0.0001
Diethyl ether	*y* = 0.0941*x* + 0.0014	0.996	<0.0001
Dichloromethane	*y* = 0.0657*x* + 0.0005	0.996	<0.0001
Chloroform	*y* = 0.0313*x* + 0.001	0.996	<0.0001
Ethyl acetate	*y* = 0.0821*x* + 0.002	0.996	<0.0001
n-Butanol	*y* = 0.515*x* + 0.0322	0.995	<0.0001

^a^Equation obtained from calibration curve. ^b^ *R* ^2^ obtained from calibration curve. ^c^ *t*-test for linear regression coefficient.

**Table 4:** Intra- and interday accuracy and precision by GC/MS.

	QC sample (*n* = 5)	Intraday		Interday	
		Accuracy (%)	RSD (%)	Accuracy (%)	RSD (%)
Ethanol	LLOQ	3.4	10.2	6.7	5.0
	LQC	7.4	−4.5	3.0	−2.0
	MQC	5.5	−5.8	5.3	−0.7
	HQC	9.2	−1.9	6.1	2.5
	DQC	8.6	−9.6	11.6	0.4

Diethyl ether	LLOQ	7.3	−12.3	7.4	−5.0
	LQC	5.1	−14	8.1	−6.6
	MQC	9.2	−3.8	11.9	−1.1
	HQC	13.0	−1.8	7.0	2.6
	DQC	13.6	0.1	5.0	0.6

Dichloromethane	LLOQ	8.9	−7.6	11.1	−10.0
	LQC	10.7	4.9	13.7	−8.3
	MQC	7.2	−6.2	10.7	−3.3
	HQC	12.2	−2.4	9.7	−2.3
	DQC	13.1	−2.7	6.1	−0.6

Chloroform	LLOQ	7.9	−6.7	7.4	−5.0
	LQC	9.6	−10.1	4.0	−12.5
	MQC	10.6	−11.3	8.2	−7.2
	HQC	13.8	−6.3	6.5	−1.3
	DQC	13.5	−4.9	2.3	−2.6

Ethyl acetate	LLOQ	8.3	−6.9	14.1	6.6
	LQC	5.3	−1.7	5.0	0.4
	MQC	7.7	−7.9	4.8	−12.7
	HQC	5.9	−3.4	9.7	−12.1
	DQC	4.9	−9.3	3.9	−12.0

n-Butanol	LLOQ	3.3	4.9	7.7	10.0
	LQC	5.7	−4.4	2.9	−2.3
	MQC	5.0	−4.8	3.7	−1.4
	HQC	8.8	−1.4	0.4	−1.5
	DQC	0.9	−10.8	6.8	−3.5

RSD: relative standard deviation, LQC: low quality control, MQC: middle quality control, HQC: high quality control, and DQC: dilution quality control.
